# Physician challenges and supports during the first wave of the COVID-19 pandemic: A mixed methods study

**DOI:** 10.3389/fpsyt.2022.1055495

**Published:** 2022-12-08

**Authors:** Myia S. Williams, Laura Ryniker, Rebecca M. Schwartz, Pooja Shaam, Kayla D. Finuf, Samantha S. Corley, Nidhi Parashar, John Q. Young, Mayer H. Bellehsen, Sophia Jan

**Affiliations:** ^1^Department of Medicine, Northwell Health, Manhasset, NY, United States; ^2^Donald and Barbara Zucker School of Medicine at Hofstra/Northwell, Northwell Health, Hempstead, NY, United States; ^3^Institute of Health System Science, Feinstein Institutes for Medical Research, Manhasset, NY, United States; ^4^Department of Occupational Medicine, Epidemiology and Prevention, Northwell Health, Great Neck, NY, United States; ^5^Center for Traumatic Stress, Resilience and Recovery at Northwell Health, Great Neck, NY, United States; ^6^Division of General Pediatrics, Steven and Alexandra Cohen Children's Medical Center, New Hyde Park, NY, United States; ^7^Department of Psychiatry, Zucker Hillside Hospital at Northwell Health, Glen Oaks, NY, United States; ^8^Department of Psychiatry and Resident Mental Health Program, Lenox Hill Hospital, New York, NY, United States

**Keywords:** COVID-19, pandemic, physician, mental health, challenges, supports, occupational health

## Abstract

**Introduction:**

During the COVID-19 pandemic, physicians encountered significant COVID-19-related negative experiences and psychological distress in both their personal and professional lives. To understand the factors that negatively impact physician well-being, a number of studies have pointed to multiple work system factors such as excessive workload and workflow interruptions. In addition, studies have shown that positive interpersonal relationships that provide social support may also serve as a buffering role against psychological distress. The aim of our study explores the challenges and sources of support for physicians relative to mental health symptoms.

**Methods:**

In this study, We used a cross-sectional study design with a convergent parallel mixed method approach combining both qualitative and quantitative data collected in parallel from a self-report questionnaire immediately following the first wave of COVID-19. The aim of our study explores the challenges and sources of support for physicians relative to mental health symptoms.

**Results:**

Of the 457 physicians in the study, the most frequently potential negative occupational experiences were, “Being at risk of contracting COVID-19 from patients/co-workers” (90.5%) and “Contact with distressed family members who cannot be with a loved one” (69.5%). We identified five common themes for main sources of social support (e.g. emotional support from family/friends) and six themes for challenges (e.g., work-related demands exacerbated by the pandemic).

**Discussion:**

Our study highlights COVID-19 and other pandemic-related challenges that negatively impacted the mental health of physicians. Interventions that provide targeted organizational supports (e.g. sufficient PPE and child support), as well as specific sources of support (e.g. family and emotional), can attenuate those challenges and stressors experienced during a pandemic.

## Introduction

The Coronavirus Disease 2019 (COVID-19) is an unprecedented outbreak that rapidly spread worldwide, with over 550 million confirmed cases and more than 6 million deaths globally as of July 2022 according to the World Health Organization (WHO). In the early stages of the pandemic millions of people around the world worked from home to contain the spread of virus, while health care workers (HCWs) faced long and tiring work shifts placing their own health at risk (1). Across various specialties and fields, all HCWs have encountered unprecedented challenges in patient care, including unknown or changing prevention and management protocols, initial shortages of personal protective equipment (PPE), rapid implementation of telemedicine, repurposed clinical areas, limited availability of in-person services, and redeployment of HCWs to unfamiliar clinical environments, (2) and psychological distress (3). Consequently, it is not surprising that many HCWs, particularly physicians, encountered significant COVID-19 related negative experiences and psychological distress in both their personal and professional lives (2, 3).

In a cross-sectional study that assessed the relationship between COVID-19-specific negative experiences and adverse mental health outcomes among HCWs, the most common concerns endorsed by physicians were fear of contagion, death of patients despite all efforts, circumstances involving contact with a distressed family member, and the inability of friends and family to understand physicians' physical and emotional exhaustion (4). Further, higher negative occupational pandemic experiences were associated with significant increases in symptoms of depression, anxiety, and PTSD (4). More specifically, other studies found that higher levels of emotional exhaustion were reported among trainee and attending physicians redeployed to unfamiliar clinical areas during the first peak of the COVID-19 pandemic (5).

As COVID-19 became a global public health crisis, it created a pivotal moment for healthcare administrators to take proactive steps and develop effective interventions to minimize the compounding effects of acute COVID-19 stressors and burnout on physician well-being. Frameworks to understand physician well-being and burnout point to multiple work system factors that contribute to clinician burnout, such as excessive workload, inadequate staffing, administrative burden, workflow interruptions and distractions, inadequate technology usability, time pressure, moral distress, and patient factors (6). These work factors stem from decisions or actions taken at various levels in health care, including local frontline care, the organizational or healthcare system, and health policy and regulatory levels (6). Factors that prevent or mitigate burnout include organizational culture, job control and autonomy, flexibility, rewards, professional relationships, social support, and work-life integration (6). Unique individual factors, such as personality (e.g., Big Five Personality traits such as neuroticism), coping strategies, and resilience, may mediate the effects of these work system factors on clinician burnout and professional well-being (6). For example, several studies investigating the effectiveness of interventions on reducing burnout have explored the relationship between personality and burnout (7). More specifically, previous studies have found that the personality trait of neuroticism is linked to an increased risk of burnout, whereas emotional stability is linked to better implementation of coping strategies and ultimately better well-being (6, 8–10). Ultimately, when designing and evaluating the effectiveness of interventions to reduce physician burnout, researchers and practitioners alike should take into consideration the factors mentioned above to ensure greater precision around intervention effectiveness for various groups of physicians (7).

In addition, positive interpersonal relationships that provide social support may also serve as a buffering role against stress. Past evidence has strongly linked adequate support from managers, co-workers, family, and friends with positive mental health outcomes for both healthcare and non-healthcare professionals during stressful and traumatic events such as accidents, natural disasters, and disease outbreaks (11). In the face of threats or stress-inducing events, social support has been shown to help individuals sustain emotional balance (12). Among HCWs, social support can help promote coping, diminish occupational stress, and prevent psychiatric symptoms and disorders (8). Quantitative studies conducted during the COVID-19 pandemic have reported similar findings. Psychological resilience, coping behaviors and social and occupational support, safeguard mental health and well-being among frontline HCWs (13, 14). For example, one study found that challenges, such as perceived lack of occupational support, was associated with two-fold increased odds of probable anxiety (4).

The COVID-19 studies to date have largely been quantitative. Taken together, the results show higher rates of anxiety, depression, PTSD, and burnout and identify important mediating factors (e.g., occupational, or interpersonal support). However, these quantitative results do not capture important elements of the physician experience. Within these categories of stressors and support, what do physicians identify as most salient to their experience? A richer understanding of the physician experience can provide, in conjunction with the quantitative literature to date, important guidance to future interventions.

The current study addresses this gap in the literature. We use qualitative methods to analyze narrative accounts of frontline physicians' experiences during the first peak of the COVID-19 pandemic. We report on the sources of social support as well as the challenges and degrees of COVID-19 related distress that frontline physicians experienced during the first peak of the pandemic.

## Methods

### Study design and participants

A cross-sectional study design with a convergent parallel mixed method approach was used (15), combining quantitative (QUAN) and qualitative (QUAL) data collected in parallel (QUAN + QUAL) (15, 16) using a self-report questionnaire from a pre-existing longitudinal registry data of HCWs across the largest not- for profit healthcare system in New York State. The purpose of this registry is to evaluate HCWs personal and occupational well-being as it relates to the COVID-19 pandemic. The registry consists of two cohorts: physicians and nurses/nursing staff. Both cohorts receive the self-report survey every 6 months for 5 years. The registry is IRB approved (#20-0510).

For the current study, we analyzed baseline data from the physician cohort. The quantitative study employed a descriptive, cross-sectional design using baseline assessments among physicians between June 21-August 21, 2020, and evaluated self-reported COVID-19 exposures from the three preceding months (March–May 2020). The qualitative study involved two open-ended questions included in the baseline assessments to explore support and challenges experienced by physicians within the same time frame. Data for both the QUAN and QUAL were analyzed independently and results from both mixed-methods strands were integrated. Quantitative measures were used to describe the sample and to contextualize the qualitative findings using a convergent parallel design (16).

We utilized a secure, HIPAA-compliant database, Research Electronic Data Capture (REDCap), for all consent and baseline assessment data collection purposes. All study measurements, including COVID-19-related exposures, potential modifiers, mental health outcomes, and open-ended questions regarding support and challenges were contained within the electronic baseline questionnaire, which was emailed directly to eligible participants. Participants eligible for study inclusion included physicians (attending, resident, or fellow) who were employed by or affiliated with the health system between March 2020 and August 2020 and able to electronically consent for participation. Exclusions included individuals who were either unable to electronically consent or not employed or affiliated during the above-mentioned time frame.

Of the 12,542 eligible physicians who were sent the baseline questionnaire, 620 physicians completed it, resulting in a 4.9% participation rate. Of this total, 457 physicians provided responses to both the quantitative and qualitative questions. While quantitative results for the broader sample of 620 have been reported elsewhere (4), here we analyzed the data from only the 457 physicians who answered both of the open-ended qualitative questions.

### Measures

Quantitative measures used in the current study include gender (male/female), race/ethnicity, partner status, trainee status (attending/trainee), redeployment (worked in an area that is not where they typically work), direct COVID-19 patient care, COVID-19-related negative occupational experiences, occupational support, use of organizational well-being resources, resilience, and symptoms of depression, anxiety, and PTSD. *COVID-19-related negative occupational experiences* was assessed using the Supplemental Healthcare Module of the Epidemic Pandemic Impacts Inventory-Brief (EPII-SHMb) (17). The EPII-SHMb item set begins with the statement, “Have you experienced the following since the beginning of the coronavirus disease pandemic?” followed by 16 items. Scores for each of the 16 items were summed (0 = no, 1 = yes; range: 0–16). An example item is: “Comforting family members whose loved one is dying or has died.” *Occupational Support* was assessed using the question, “On a scale of 1–5, how often did you feel supported at work” with scores dichotomized by those who feel somewhat or completely supported vs. neutral or not supported. *Use of organizational well-being resources* was defined as whether a participant reported that they accessed any programs or resources that focused on well-being that were provided by the organization. *Resilience* was assessed by the Brief Resilience Scale (BRS). The BRS is a 6-item scale that assesses one's perceived ability to “bounce back” after stressful or difficult experiences. Each item on the BRS has responses between 1–5 (1 = *Strongly disagree* to 5 = *Strongly agree*). Items were averaged to create the resilience score (range: 1–5), with higher scores signifying higher levels of resilience. *Anxiety and depression symptoms* were measured using the Patient Health Questionaire-4 (PHQ-4) (18). PHQ-4 anxiety and depression subscales have separate ranges of 0–6 with a score of 3 or greater defined as probable anxiety or depression *PTSD* was assessed employing the 5-item Primary Care PTSD Screen for the DSM-5 (PC-PTSD-5) (19). The PC-PTSD-5 contains five yes/no questions, with any positive endorsement corresponding to 1-point, and any negative endorsement resulting in 0 points for each question. All positive responses are summed to a total score, with a range of 1 to 5. Scores of 3 or higher were suggestive of probable PTSD (19).

Qualitative data were collected from two open-ended questions on sources of support and challenges faced during the time frame described above. To assess support, participants were asked: “*In the last 6 months, what has been the most important sources of support for any work-related stress you have experienced? What helped you the most?”* To assess challenges, participants were asked*: “In the last 6 months, what has been the biggest work-related challenge you have faced?”*

## Analyses

### Quantitative data analyses

Descriptive statistics (e.g., mean/SD for continuous variables and frequency/percentage for categorical variables) were used to describe the demographics of the participants as well as other relevant quantitative measures such as occupational stressors, resilience, probable PTSD, probable depression, and probable anxiety (see Table 1). All quantitative descriptive analyses were performed through IBM SPSS v28 (20).

**Table 1:** Demographics and descriptive statistics of study sample (*N* = 457).

**Variable**	***N***	%^*^
	**Mean (SD)**	
Age (Range: 20–81)	47.23 (13.0)	
**Gender**		
Female	239	52.4
Male	215	47.1
Prefer not to answer	2	0.4
Race^**^		
White	333	72.9
Black/ African American	19	4.2
American Indian / Native Alaskan	1	0.2
Asian	83	18.2
Native Hawaiian / Pacific Islander	0	0
Other	27	5.9
**Hispanic Ethnicity**		
No	401	89.5
Yes	47	10.5
**Marital Status**		
Single	75	16.5
Engaged	14	3.1
Married	327	71.9
Separated	2	0.4
Divorced	26	5.7
Widowed	7	1.5
Other	4	0.9
**Redeployed**		
No	338	74.0
Yes	119	26.0
**Did you directly care for patients with COVID-19 or suspected of having COVID-19?**		
No	96	21.0
Yes	361	79.0
**Trainee Status**		
Attending	374	81.8
Trainee (Resident/Fellow)	83	18.2
**On a scale of 1–5, how often did you feel supported at work?**		
1. Not supported	14	3.1
2. Somewhat not supported	20	4.4
3. Neutral	66	14.5
4. Somewhat supported	161	35.5
5. Completely supported	193	42.5
Number of individuals < 18 years who live in your household (Range: 0–6)	0.96 (1.2)	
Number of individuals > 65 years who live in your household (Range: 0–3)	0.30 (0.7)	
EPII Score (Range: 0–16)	7.11 (3.5)	
Brief Resilience Score (Range: 1–5)	3.89 (0.8)	
**Depression**		
No	402	88.7
Yes	51	11.3
**Anxiety**		
No	370	81.5
Yes	84	18.5
**PTSD**		
No	347	77.5
Yes	101	22.1

* Valid percents were used and do not include missing data in the calculation.

** Collected as a 'check all that apply' question, therefore percentages add to over 100%.

### Qualitative data analysis

Responses to open-ended questions were analyzed using thematic analysis (21). Responses to open-ended questions were read multiple times independently by three authors, XX, XX, and XX to ensure familiarity with the data and to get an understanding of the content of the experiences of the physicians. Each author separately documented initial theoretical and reflective thoughts, as well as potential codes and themes for challenges and social support. This process was guided by the aim of the study to determine the table 19 pandemic. Initial codes and themes were coded in Microsoft Excel and then analyzed in NVivo 12 (22). All potential themes were discussed by the three researchers collectively and revised when necessary. Once a consensus was reached among the three authors, the themes were then brought to the larger group encompassing all authors. Any disagreements between the initial three authors were rectified through discussion and revised until consensus was reached by all authors.

### Mixed-method analysis

To provide a richer understanding of the sources of support and challenges experienced by physicians during the COVID-19 pandemic, we integrated the QUAN and QUAL data following a convergent parallel design (23). We used a *narrative staged approach* for reporting and integrating our mixed-methods results (24). We present the QUAN results, then the QUAL results and end with the integration of the two.

## Results

### Quantitative results

Of the 457 physicians in the study, the mean age was 47.23 (SD = 13.0) (Table 1). The majority were White (72.9%) and non-Hispanic (89.5%). About half were female (52.4%). These demographics generally reflect those of physicians in the larger health system. Mean EPII score was 7.11 (SD = 3.5). Table 2 presents the frequencies of each EPII item representing each potential negative occupational experience. The most frequently endorsed items were: “Being at risk of contracting COVID-19 from patients/co-workers” (90.5%); “Contact with distressed family members who cannot be with a loved one” (69.5%); “Comforting family members whose loved one is dying or has died” (60.4%); and “Deaths of patients despite heroic efforts by the treatment team” (59.8%).

**Table 2:** Participant endorsement of EPII SHMb items 1–16.

**EPII-SHMb item**	**Participants who endorsed the item**	
	***n***	%^*^
Being at risk of contracting COVID-19 virus from patients or co-workers.	412	90.5
Contact with distressed family members who cannot be with a loved one.	316	69.5
Comforting family members whose loved one is dying or has died.	276	60.4
Deaths of patients despite heroic efforts by the treatment team.	271	59.8
Illness and uncertain recovery, or deaths, of co-workers.	250	54.8
Family and friends don't understand the emotional and physical exhaustion caused by your work.	227	50.0
Insufficient/unavailable viral infection testing kits.	214	47.0
Stigma from others because of your (actual or perceived) coronavirus exposure as a healthcare professional/worker (“treated like we're lepers”).	186	40.8
Having no break from disruptive noise and wearing uncomfortable PPE.	182	40.1
Family and friends don't understand the danger you face at work.	164	36.0
Insufficient staffing or equipment to properly care for COVID-19 patients.	163	35.9
Inadequate/unhygienic personal protective equipment (PPE).	153	33.7
Forced separation from your children or spouse/partner for a week or more due to work or self-quarantine.	114	25.0
Co-workers treat you or each other with irritability, impatience, or disrespect.	113	24.8
Being in close contact with patients without adequate PPE.	110	24.1
Insufficient support from workplace supervisors or administrators.	92	20.2

* Valid percents were used and do not include missing data in the calculation.

### Qualitative results

#### Support

We identified five main themes for sources of support: (i) emotional support (e.g., family and friends), (ii) informational support, (iii) appraisal support (e.g., organizational, and professional identity), (iv) utilization of mental health support resources, and (v) intrapersonal support (e.g., cultivation of a resilient mindset (see Table 3).

**Table 3:** Themes for support.

**Theme**	**Sample quotes**
Theme 1: Emotional Social Support	“*My wife and children were the most impactful support for me during the pandemic. Their ability to listen, comfort, provide a hug (while wearing a mask!) was incredibly helpful.” “My colleagues in the Division of XXX provided great emotional and technical support throughout. The internal dynamic of the group is of total mutual support.”*
Theme 2: Informational support	“…*Also the constant & ongoing educational updates by the Health Care System experts aided us in caring for our patients*…” “*At XXX Medical group, weekly group phone calls to give us update on testing, guidelines, etc., were extremely helpful. E-mails with updates also very helpful.”*
Theme 3: Appraisal support	“*The hospitalist team. The camaraderie of the group and their willingness to take me through every clinical situation and EMR question.” “…The support from people inside and outside the hospital as they donated items/food- It made me feel appreciated. Also, getting/ preparing food was one less thing I had to worry about…”*
Theme 4: Well-being support	“*I was seeing a therapist prior to the pandemic and have continued to see one. It has been absolutely vital”. The free Headspace subscription is excellent because I think meditation helps a lot…”*
Theme 5: Intrapersonal support	”*… spiritual belief made me even stronger and positive person” I was very grateful for the [employer-sponsored childcare benefit] care money because I was able to give that to my sisters.”*

#### Theme 1: Emotional social support

Emotional support was the most cited support type that respondents indicated. Emotional support from family and friends (*n* = 153) aided physicians in coping and navigating through the turmoil and chaos caused by the pandemic. For example, one participant wrote the following: “*My wife and children were the most impactful support for me during the pandemic. Their ability to listen, comfort, provide a hug (while wearing a mask!) was incredibly helpful.”* Similarly, it was important to respondents that they were able to connect with others who shared similar experiences and with whom they could discuss their feelings and concerns. Several participants reported receiving support from co-workers (127). A participant wrote:

“*My colleagues in the Division of XXX provided great emotional and technical support throughout. The internal dynamic of the group is of total mutual support.”*

In addition, participants also reported receiving a significant amount of organizational support (101) (e.g., leadership). One respondent reported: “*Our leadership was present and accessible. They kept us up to date on treatment protocols as much as possible. They showed that they cared about our safety by making changes in how we evaluate COVID patients. The scheduling was flexible and other responsibilities (e.g., billing, queries) were decreased.”* Lastly, respondents also reported receiving support from healthcare groups on social media (*n* = 10) platforms, such as Twitter. One participant wrote: “*Utilized a Twitter advertised healthcare group therapy session 1-2x/week at times I really needed it. Also, my med twitter community is superb in providing away from work conversation and support.”*

#### Theme 2: Informational support

This kind of support came via frequent dissemination of educational resources and knowledge on information regarding COVID-19 from legitimate and trusted sources [e.g., organizational updates (n = 65), updates in science (*n* = 11), information from government leadership (*n* = 7), and updates sent *via* professional affiliations (*n* = 6)].

In the early stages of the pandemic, information on COVID-19 was rapidly changing which created an additional layer of stress for physicians in treating and providing care to their patients. Informational support from legitimate and trusted sources provided physicians with instructional guidance on the “dos and don'ts” around treating patients, workplace safety, and stopping the spread of the virus. The informational support also encouraged them to explore appropriate coping responses that helped counter the perceived lack of control and helplessness experienced during the pandemic due to a lack of or conflicting information. For instance, one physician indicated,”…*Also the constant & ongoing educational updates by the Health Care System experts aided us in caring for our patients*…”

Physicians also reported welcoming informational support from government leaders such as former governor of New York, Andrew Cuomo and Chief Medical Advisor to the President, Dr Anthony Fauci. A respondent wrote, “*The updates from XXX Health System, the discussions on Zoom and the daily updates by Andrew Cuomo….Fauci…”* Another physician wrote about professional affiliations, “*At XXX Medical group, weekly group phone calls to give us update on testing, guidelines, etc., were extremely helpful. E-mails with updates also very helpful.”*

#### Theme 3: Appraisal support

Another source of support provided to physicians that was particularly important is appraisal support, which refers to the provision of feedback regarding one's performance or personal qualities or other sources of information that is relevant to self-evaluation (e.g., outside recognition and acknowledgment of being a hero) (25, 26). This type of support encompasses various affirmations that ground physicians in their identity and appropriateness of acts of service or profession that reduce uncertainty and help physicians cope with the COVID-19 pandemic (27, 28). Strong identification with the organization (hospital or medical unit) and professional identity as a caregiver provided solace for respondents. Most common forms of appraisal support were a sense of camaraderie (*n* = 40), identifying as a caregiver (*n* = 16), receiving outside recognition (*n* = 7), and identifying with the organization (*n* = 4).

Several physicians reported the camaraderie experienced within their respective groups and being able to talk to others about shared experiences provided them with a sense of “we are in this together.” Specifically, one physician said, “*The hospitalist team. The camaraderie of the group and their willingness to take me through every clinical situation and EMR question.”*

Some reported tapping into their professional identity to re-affirm the commitment to helping patients during this difficult time. For example, one physician wrote, “*My belief is that as a medical professional I am morally obligated to help my patients.”*

Similarly, outside recognition was another key form of support that helped physicians cope during this difficult time. Physicians reported being recognized, appreciated, and celebrated as a hero by the general public and respective organizations, as well-being honored with financial and food donations. According to one physician*, “***…***The support from people inside and outside the hospital as they donated items/food- It made me feel appreciated. Also, getting/ preparing food was one less thing I had to worry about…”*

Lastly, a few physicians also reported that they were proud to work for their organization. One stated, “*I was proud to be a part of this Health system because of the dynamic caring manner that they presented in TV/News/Public appearances.”*

#### Theme 4: Well-being support

Well-being support were tangible supports that physicians utilized to combat or cope with the increased pressure and detrimental effects of the pandemic on well-being. This form of support included therapy (*n* = 12), support groups (*n* = 7), exercise (*n* = 4), and well-being applications (n = 4).

Physicians noted how important it was to either seek therapy or maintain their existing relationships with their therapist to receive support/resources to cope with challenges of COVID-19 pandemic. One physician wrote, “*I was seeing a therapist prior to the pandemic and have continued to see one. It has been absolutely vital.”* Some physicians reported that support groups provided them with safe therapeutic spaces and a form of community with others going through similar experiences as “*the ability to have “safe” places to share without fear of retribution or “fallout.”*

A few physicians also indicated the importance of establishing and or maintaining daily exercise routines such as “*going outdoors every day to exercise.”* Other physicians reported making use of well-being applications like Headspace or Joyable which were freely available to all organizational members. One physician stated,” …*The free Headspace subscription is excellent because I think meditation helps a lot…”*

#### Theme 5: Intrapersonal support

Over time, individuals acquire and utilize protective factors from other individuals and resources around them. Specifically, individuals can utilize social support, tap into their existing resources, and seek help to mitigate the harmful effects of adversity. For physicians, intrapersonal support came from religiosity and spirituality (*n* = 9), and gratitude (*n* = 5).

Relying on their faith, religious customs, and practices (e.g., praying the Rosary), strong spiritual upbringing, and belief in humanity provided physicians with a sense of safety and gave them the ability to carry on each day during the pandemic. One physician wrote*,”… spiritual belief made me even stronger and positive person.”* Additionally, knowing that others were concerned about their well-being, financial and other forms of support received from the organization, and honoring of human resource policies by the organization (e.g., vacation times) provided a sense of gratefulness that gave physicians the “push they needed to move forward” despite the toll of the COVID-19 pandemic. A respondent claimed, “*I was very grateful for the [employer-sponsored childcare benefit] care money because I was able to give that to my sisters.”*

#### Challenges

We identified six main themes for challenges, namely (i) COVID-19 specific stressors, (ii) work-related demands exacerbated by the COVID-19 pandemic, (iii) worked-induced psychological distress, (iv) death and dying, (v) child-care demands, and (vi) additional stressors associated with the “new normal” (see Table 4).

**Table 4:** Themes for challenges.

**Theme**	**Sample quotes**
Theme 1: COVID-specific stressors	“*Coping with the idea that people I knew were going to get it and the uncertainty of them surviving. All I kept thinking was “there's no way I'm gonna get through this unscathed.”* “*Not being able to test for COVID infection in the office and not knowing what to look for in terms of symptoms*
Theme 2: Work-related demands exacerbated by the COVID-19 pandemic	“*So much administrative work and balancing with deployed staff and needing to provide patient care and all the family obligations and responsibilities. It was absolutely exhausting. I think women really took the brunt of this pandemic and I'm exhausted and there is no break on the horizon.” “I felt we had inadequate PPE and were being asked to reuse things that were marked as single use or disposable with no credible research to support that it was safe… I felt my safety was jeopardized on a daily basis… I considered quitting my job multiple times because I felt it was so unsafe at work… It was a horrific experience…”*
Theme 3: Worked-induced psychological distress	“*My husband has been afraid to live with me despite my relatively low risk of exposure until about 1-2 weeks ago. Not being able to socialize or travel to visit with my daughter. Social isolation generally.” “Fear of getting myself or my family sick not knowing when it is going to end. Emotional exhaustion and trauma of multiple sick and dying patients. Isolation from my friends and family.”*
Theme 4: Traumatic stress associated with pandemic related death and dying	“*The loss of life was the most difficult challenge. We lost so many patients. I recall their names and at times will well up with tears thinking about their death and their families “Fear of losing parents and in-laws. My possible death and partner's possible death and possible loss of parenting 6 y/o son”*
Theme 5: Childcare demands	“*The biggest challenge was taking care of patients and then coming home and flipping to the roll of mother and teacher. That has given me tremendous stress. The medicine I could deal with. The double stressor is what made it unbearable at times.” “I am a single parent, and the childcare situation is frightening, I don't want to put my kids into an exposed situation, and I don't know how to work and make sure they are okay. Having options for leave or temporarily reduced schedules is critical to me.”*
Theme 6: Additional stressors	“*Concern about losing my job or being furloughed since there was not much demand for dermatology during the height of the pandemic.” “Adjusting to the technology of telehealth but dealing with the feelings that I was not adequately caring for people who needed more”*

#### Theme 1: COVID-specific stressors

COVID-specific stressors were the most cited challenge respondents reported. Collectively, these stressors were demanding events or stimuli that were not present prior to but rather developed because of the COVID-19 pandemic and related deaths. Specifically, these stressors stemmed from uncertainty regarding proper treatment (*n* = 45), generalized uncertainty (*n* = 43), fear of contracting COVID (*n* = 41), an inability to properly treat (*n* = 41) or test (*n* = 12) patients with COVID, and concerns about transmitting the virus to loved ones (*n* = 26).

Traditionally, physicians employ evidence-based approaches to diagnose and treat patients. These approaches are developed over years of practice and research and provide physicians a certain level of guidance, comfort, and assurance with which they treat their patients. However, since COVID-19 was a new, dangerous, and widespread pathogen with no published literature nor collective clinical experience, COVID-19 soon become a disease associated not only with high mortality, but also with a high degree of uncertainty in its etiology and management, which left physicians with significant stress and challenges. As one physician stated: “*Unable to provide concrete medical advice to patients and families. I am used to evidence based medical guidelines and clear concise approaches to medical issues, but the virus did not have a clear protocol as we were trying to figure out what worked and what didn't. It felt like I was doing a clinical trial without consent, but it was using the information available.”*

In addition, some physicians also reported feeling generalized anxiety about the entire COVID-19 pandemic as it related to what would happen to themselves, patients, families, and life as we know it. Another physician noted, “*Coping with the idea that people I knew were going to get it and the uncertainty of them surviving. All I kept thinking was “there's no way I'm gonna get through this unscathed.”*

Physicians also stated that there was a fear of contracting COVID-19, particularly because of their own underlying medical conditions, as well as the consequences of contracting the virus (e.g., impact on family). One physician revealed*, “Fear of getting infected, getting my loved ones infected, dying and leaving my family/children unprotected.”*

Physicians also reported challenges with properly treating COVID-19 patients, especially since there was a dearth of treatment protocols and medication on how to care for patients. One response said: “*Not being able to save a lot of people no matter what we did. Knowing that as I'm intubating someone, there's a very high chance that they wouldn't make it. It didn't feel as if I was killing them, more like signing their death certificate in front of them while they are alive…. That's part of the job, but we typically aren't doing it multiple times a day.”*

Similarly, there were challenges with testing, including insufficient tests, frequent changes in testing protocols, testing delays, or testing family members of patients (e.g., parents of hospitalized children) with long hospitalizations ahead, particularly those at high-risk for complications (i.e., pregnant family members). A physician wrote, “*Not being able to test for COVID infection in the office and not knowing what to look for in terms of symptoms.”*

Lastly, some physicians also feared contracting the disease and transmitting it to their loved ones. Physicians also reported that this fear was also compounded by loneliness from self-imposed isolation from family and friends, fear of putting loved ones at risk of death, and inability to hold and be with family. Most physicians had similar fears as this physician who wrote: “*For weeks at the beginning, I was afraid that my wife and my family would contract Covid-19 from me and fare poorly. I was constantly concerned for my colleagues who were sent to work in the Covid-19 units.”*

#### Theme 2: Work-related demands exacerbated by the COVID-19 pandemic

Aspects of the working environment also imposed significant challenges on the ability of physicians to perform their work duties seamlessly. Unlike the aforementioned COVID-specific stressors, work-specific stressors may have been present prior to the pandemic and were exacerbated by it. Physicians who indicated these challenges reported increased workload (*n* = 57), administrative demands (*n* = 34), lack of personal protective equipment (PPE; *n* = 32), issues surrounding deployment (*n* = 16), internal conflict (*n* = 9), and conducting research (*n* = 5). Physicians reported that already high workloads were even higher:

“*So much administrative work and balancing with deployed staff and needing to provide patient care and all the family obligations and responsibilities. It was absolutely exhausting. I think women really took the brunt of this pandemic and I'm exhausted and there is no break on the horizon.”*

Other physicians expressed the impact of pandemic-imposed measures, policies, and institutional-level decisions by the administration and organizational leadership as a significant challenge affecting the ability to perform work duties adequately: Specifically, one physician wrote:

“*Having to follow the demands of administration who don't work in the clinical inpatient setting and have no true understanding of the hospital. They are completely disconnected and unattached to the people that work in the hospital, yet they dictate who works, how long they work and when they work. It felt irresponsible to have administrators who sit behind desks all day managing people who are actually caring for patients.”*

Availability of PPE was also a challenge considering that fear of contracting COVID-19 and transmitting it was a significant fear for physicians. A lack of PPE as well as protocols on its use had a profound impact on both physicians' ability to do their job safely and their psychological and physical well-being. A physician added:

“*I felt we had inadequate PPE and were being asked to reuse things that were marked as single use or disposable with no credible research to support that it was safe… I felt my safety was jeopardized on a daily basis… I considered quitting my job multiple times because I felt it was so unsafe at work… It was a horrific experience…”*

Deployment was an important factor in helping combat the COVID-19 pandemic and reducing strain on the physician workforce. Physicians believed that many deployment-related decisions were not executed properly, in terms of schedules, communication and transparency of decisions among other things:

“*Redeployment in our division. 2/3 of our division members called upon, told 2 weeks, kept for 6….Those deployed are variably burned out, upset with us for less than stellar communication with them while they were at hospital. Leadership in our division had little idea how to strategize our redeployment lists because there was little week to week communication from redeployment leadership.”*

Moreover, collaborative efforts and policies for conducting research were described as challenging and in some cases inefficient:

“*I am very distraught about how research was handled during the pandemic. We were prevented from pursuing clinical research (that would not have incurred a cost or resources) but the “research group” locked up the IRB and held proposals in queue, only approving a maximum of 5 per week. Given there were 500*+ *in queue, it would take several years to clear the list, when the topic would no longer be relevant. Not all departments were asked to participate and there was no general notice to include interested participants.”*

#### Theme 3: Worked-induced psychological distress

Physicians reported feelings of isolation (*n* = 55), helplessness (*n* = 26), and emotional exhaustion (n = 25). Physicians noted the extended periods of time where they were unable to physically be with loved ones took a toll on their well-being. Additionally, respondents noted their profession was centered on caring for others, but they were unable to do this which resulted in interpersonal feelings of helplessness, hopelessness, and despair. One physician claimed:

“*My husband has been afraid to live with me despite my relatively low risk of exposure until about 1-2 weeks ago. Not being able to socialize or travel to visit with my daughter. Social isolation generally.”*

Lastly, physicians reported being emotionally exhausted:

“*Fear of getting myself or my family sick not knowing when it is going to end. Emotional exhaustion and trauma of multiple sick and dying patients. Isolation from my friends and family.”*

#### Theme 4: Traumatic stress associated with pandemic related death and dying

At the height of the first wave of the COVID-19 pandemic, physicians were exposed to high volumes of death on a daily basis; the volume of end-of-life decisions imposed a significant burden to their psychological well-being. Physicians reported the sheer volume of death (*n* = 25), moral distress (n = 9), and death of a family member or loved one (*n* = 8) as significant challenges related to their practicing during the pandemic. Witnessing numerous deaths in a single day and concurrently being unable to help or save patients caused extreme stress for respondents. This phenomenon was cited as a contributing factor to their emotional and physical well-being, leaving them more vulnerable to adverse mental health outcomes:

“*The loss of life was the most difficult challenge. We lost so many patients. I recall their names and at times will well up with tears thinking about their death and their families*

The rising COVID-19 associated morbidity and mortality left physicians with morally challenging and distressing end-of-life decisions, such as “*having to make decisions and potentially lifesaving decisions with limited supervision.”* Alongside moral distress, physicians were also faced with the fear and/or possibility of death of both themselves as well as family members or loved ones and the impact this would have on future finances and family dynamics:

“*Fear of losing parents and in-laws. My possible death and partner's possible death and possible loss of parenting 6 y/o son”*

#### Theme 5: Childcare demands

Physicians noted demands associated with having to care for children at home while also being deployed during the pandemic. These challenges stemmed from role conflict (*n* = 23) and issues surrounding general childcare (*n* = 14). Physicians indicated feelings of being inadequate and unequipped with childcare, specifically issues regarding remote learning.

Physicians faced conflicts between working pressures and family roles. Prior to the COVID-19 pandemic, there was some sort of separation between work, family, and school. Stay-at-home orders and remote learning blurred that separation. Physicians indicated that the workload and demands from the COVID-19 pandemic prevented them from concentrating and balancing important things in their family lives (e.g., assisting with remote teaching/learning), hence there was a feeling that work undercut their capacity to perform home and child-care related roles. According to one physician who experienced role conflict:

“*The biggest challenge was taking care of patients and then coming home and flipping to the roll of mother and teacher. That has given me tremendous stress. The medicine I could deal with. The double stressor is what made it unbearable at times.”*

Similarly, physicians also reported challenges surrounding general childcare:

“*I am a single parent, and the childcare situation is frightening, I don't want to put my kids into an exposed situation, and I don't know how to work and make sure they are okay. Having options for leave or temporarily reduced schedules is critical to me.”*

#### Theme 6: Additional stressors

A small percentage of physicians indicated additional stressors such as financial and/or economic stressors (*n* = 18), the emergence of conspiracy theories surrounding COVID (*n* = 16), issues regarding the use of telehealth (*n* = 9), and challenges associated with health disparities (*n* = 3). For some physicians their clinics or practices were forced to halt, creating concerns around money and loss of income. While many clinics or practices rapidly implemented telehealth, a lack of existing telehealth infrastructure and telehealth training made using the new technology challenging, both for physicians and patients. Additionally, the rapid spread of misinformation and pseudoscience, and growing publicly held beliefs that the pandemic was faked, created tension for physicians, particularly when dealing with friends or loved ones who perpetuated these beliefs.

Finances are considered an important factor for positive psychological well-being, yet there is overwhelming evidence that the COVID-19 pandemic negatively impacted the financial lives of many individuals, including physicians. This was partially a result of government-imposed lockdowns, closures of practices due to low demand, patient fears of visiting medical offices, and mandated health system pause in non-emergent surgeries. As one dermatologist stated, “*Concern about losing my job or being furloughed since there was not much demand for dermatology during the height of the pandemic.”*

While there was initially a lack of published medical information about the COVID-19 virus, there was a preponderance of misinformation and conspiracy theories surrounding COVID. Physicians noted how challenging it was that family members did not believe them, instead believing the conspiracy theories or not taking the threat of COVID seriously:

“*It's infuriating that some people are making a show of not wearing a mask. They don't see what happens in the hospital, and until it happens to their loved ones, it's a hoax to prevent them from getting haircuts. It's extremely depressing that you just can't fix stupid people.”*

With the move to the “new normal” of being remote and a reduction in non-emergent surgeries and office visits, physicians had to quickly become proficient in using telehealth technology to diagnose and treat patients. Physicians reported the challenges of rapidly moving to telehealth as well as the issues surrounding technology, without the assistance of proper physical examination and tests to aide them:

“*Adjusting to the technology of telehealth but dealing with the feelings that I was not adequately caring for people who needed more”*

Lastly, COVID-19 pandemic brought into the forefront long-existing health disparities and systemic injustices in healthcare. A few physicians reported that it was challenging seeing those inequities play out, particularly “*seeing so many people of color singled out by this disease.”*

### Integration of quantitative and qualitative results

The most frequently endorsed negative occupational experiences captured by the EPII in the quantitative results reflect aspects captured by the first theme under challenges described in the qualitative results, “COVID-19 specific stressors.” The mechanism by which these negative occupational experiences created poor well-being was due to the high degree of uncertainty around etiology and management of a new illness with a high mortality, compounded by lack of sufficient supplies for testing and protection, leading to generalized feelings of fear, anxiety, and frustration. The quantitative results did not describe the full array of challenges experienced by physicians, nor did it describe sources of support described in the qualitative results.

## Discussion

This study used a convergent parallel mixed-methods approach to gain a comprehensive understanding of the sources of support and challenges faced by physicians during the COVID-19 pandemic. Together we compared and contrasted both sources of data for evidence of convergence and divergence. In summary, our quantitative study indicated high distress with little explanation regarding the context of the occupational stressors. However, the qualitative analysis gives us a much richer understanding of the sources of support and challenge. Taken together–we have a picture that is more complete and provide possible implications that are more specific than with quantitative alone.

Our qualitative analysis indicated that physicians' most common source of support to cope with the COVID-19 pandemic was family and friends. However, where the findings diverge somewhat from the quantitative data is in the fact that 50% of the physicians felt that friends and family do not understand the exhaustion caused by their work. Our results are consistent with previous studies that show that married HCWs and those with children reported decreased odds of stress, burnout, anxiety, and depression during the COVID-19 pandemic (4, 29–31). In our study, about 75% of the sample was in a relationship, however, the quality of that relationship in terms of support provision is unknown. Providing additional context for our qualitative findings in which those that reported high levels of organizational and coworker support felt that this type of support was key to coping with the pandemic while at work (32–34). However, less than a quarter of participants utilized this support. Our results are consistent with other studies that show that both organizational and coworkers' social support reduces the negative impact of occupational stressors and prevents common psychological strains (e.g., anxiety) during the COVID-19 pandemic (4, 35, 36). Moreover, research shows that such support is significant for physicians as it affects self and professional efficacy (27, 28, 37). Our quantitative data indicated that about 78% of physicians felt supported at work, so the qualitative data regarding reliance on co-worker support is not surprising.

It has well been documented those positive interpersonal relationships help decrease burnout and stress while improving the physical and psychological well-being of physicians (32–34). One qualitative study on critical care pediatric physicians found that at least 70% of participants reported positive interpersonal relationships, that is, being able to count on co-workers and leadership teams for support during a crisis, as a significant factor in reducing burnout (32). Similarly, a study on the impact of a psychological resilience intervention (Battle Buddies model of peer support) on healthcare workers during the COVID-19 pandemic, researchers found that positive interpersonal relationship fostered social connectedness among Battle Buddies (healthcare workers going through the same experiences) which in turn helped to reduce burnout and effective implementation of coping strategies. This is because HCWs experiencing similar stressors were able to lean on each other for support thereby promoting emotional validation, normalization of the traumatic experience of the pandemic, sharing of resources and problem solving. This in turn resulted in feelings of hopefulness, comradery, compassion, gratitude, and safe space to share positive emotions (33). During the COVID-19 pandemic having a community of interpersonal social support provided physicians with the resources needed to cope with the demands of COVID-19, attenuate strong emotions, and reframe their traumatic experiences while at the same time fulfilling their need for belonging, relatedness and building self-efficacy all of which are key factors in coping with and reducing burnout and increasing psychological well-being (33, 34).

Our qualitative results also support the idea that physicians relied on multiple sources of coping, including emotional support from coworkers and family, informational support, appraisal support, and mental health support, all of which help to build resilience (26, 30, 38, 39). The qualitative results regarding mental health support are consistent with previous studies focused on coping strategies for HCWs during the COVID-19 pandemic (30, 39). Specifically, one study from New York found HCWs engaged in mental health resources, such as talk therapy and support groups, as well as exercise and religious/spiritual practices as part of their stress-reduction activities (39).

Further, our qualitative results show that other than emotional social support, informational support from educational and evidence-based COVID-19 information from legitimate and trusted sources was an important coping mechanism for physicians. While the relationship between informational support and physicians' mental health in a pandemic context has not yet been established (40), our data support previous findings where informational support as an organizational resource is a significant but distinct influence on how employees cope with a lack of information and support organizational decisions during a pandemic (40). Results show that informational and relational communication as organizational resources have a significant but distinct influence on how employees support their employer during a crisis. Importantly, our findings in our mixed-method study highlight the need for rapid interventions that are inclusive of both informational and emotional social support resources. Thus, although physicians can tap into their social networks for emotional social support, they also need resources that provide helpful and instructive information on how to treat patients (40). Organizations should recognize the importance of informational support in their physician well-being strategy (37). This is further bolstered by the quantitative findings regarding specific items within the EPII that indicate that between 33–47% of physicians experienced lack of necessary PPE, equipment, and viral testing kits. Although these items are not informational, *per se*, these issues are tied to the initial lack of knowledge about the virus and translated into not having the resources needed to best respond.

Several physicians reported that they were highly concerned with keeping themselves, their loved ones, and their patients safe, while simultaneously providing the most up-to-date care for treating the virus. Both our quantitative and qualitative data found that the most endorsed occupational stressor was being at-risk of contracting COVID-19 (90%). The amount of information presented to the physicians *via* news coverage and organizational announcements was overwhelming, resulting in a sense of personal and general uncertainty. Physicians also reported that challenges related to reducing the risk of transmission of COVID-19 to family, higher workload and workplace demands created by institutional level COVID-19 policies, shortages of PPE, compounded by perceived helpless and anxiety and depression from social isolation as major factors adversely impacting their mental health. Moreover, the sheer volume of patient death and needing to make end-of-life and split-second decisions without sufficient information were seen as morally challenging and distressing for physicians. In fact, the quantitative data indicated that 60% of the physicians described witnessing the death of patients despite heroic measures from the treatment teams. This is consistent with recent studies that explore predictors of the adverse psychological impact of COVID-19 experiences on physician psychological and physical well-being (30, 37, 41, 42).

While the occupational challenges of the COVID-19 pandemic among physicians are more frequently described in the literature, we found that non-occupational stressors such as childcare demands, financial insecurity, and racial disparities, though similar to general employee contexts, were also important factors for physicians (30, 43). Specifically, compared to members of the general public under stay-at-home orders, the additional burden of providing patient care and increased administrative workloads left physicians struggling with child-care responsibilities particularly since children were engaged in remote learning. This situation was even more relevant for single parents and dual households where both parents worked in healthcare. Similarly, physicians also experienced role conflict with being concurrently a physician, parent, and teacher. The quantitative data highlights an additional potential caregiving burden in that 25% had to separate or quarantine from their family members due to potential fears around COVID exposure. Our findings are consistent with previous studies that show adverse mental health are linked to role conflict, work-family conflict, and reduced work-life balance among physicians (30, 37, 44, 45). While this is beyond the scope of our present study, future studies should explore the role of gender and intersectionality of race on the impact of COVID-19 and role conflict and child-care demands (44, 46). Previous studies have found apparent gender differences in the prevalence of certain kinds of mental health difficulties among HCWs, such that female HCWs have almost twice the risk of depression, anxiety, and posttraumatic stress disorder (PTSD) as well as a 41% higher risk of insomnia than male HCWs (47). The same should be explored for the emergence of our racism as an additional challenge for physicians. While our demographics in this study are reflective of large healthcare systems, we have too few under-represented minorities in medicine to conduct secondary analyses.

Finally, our study identified specific additional structural challenges, which may also be potential intervention targets by health care employers and managers. Physicians reported that pandemic-related measures such as lockdowns, furlough, and pause on non-emergent medical procedures also posed significant challenges in terms of job and financial insecurity. A mixed-method study found that physicians in Jordan reported these same measures as having impacted their psychological and physical well-being. Future interventions should investigate financial incentives and support as appreciation for physicians' efforts (37). Quotes from our support sources show that physicians felt appreciated and expressed gratitude for financial donations provided both internally (e.g., childcare bonus) and externally (e.g., donations from the Chinese community). Despite these structural issues, only 20% of physicians reported feeling insufficient support from workplace supervisors or administrators which, although not as low as would be preferred, was the least frequently endorsed item on the EPII.

Strengths of our study include (i) our mixed methods approach provided us with a comprehensive understanding of physician experiences during COVID-19 both from a qualitative and quantitative perspective., (ii) our mixed methods also provided triangulation between both the qualitative and quantitative methods (37). Nevertheless, limitations of this study include a relatively low overall survey response rate, which may limit generalizability of the themes identified in this study. We are also unable to quantify the relative impact of each of the supports and challenges identified since this was primarily a qualitative study. The study participants had few individuals considered underrepresented in medicine. While the racial breakdown of our participants appears consistent with that of the greater health system, the low overall numbers of physicians who are underrepresented in medicine may limit generalizability to physicians who do not identify as White or Asian, and who were undoubtedly disproportionately affected by the pandemic (48). Finally, the study took place and explored experiences of physicians at the peak of the COVID-19 pandemic prior to the availability of home-based testing, vaccines, new therapeutic options (i.e., monoclonal antibody or Paxlovid), and wide availability of PPE. Consequently, COVID-specific occupational stressors may now look different from those during the first wave. Still, findings described here can still be valuable lessons for potential future pandemics of new pathogens or new COVID-19 variants resistant to current vaccines, testing, or therapeutics. Future studies should continue exploring the protective role of resilience and organizational support to identify interventional targets that address mental health outcomes of physicians. Future studies should also include the trajectories and longitudinal mental health impacts of physicians and other HCWs at the front lines of the COVID pandemic, particularly nurses, who typically have greater direct patient care involvement than physicians, and physician trainees, who have been shown to be particularly vulnerable to COVID-related stressors (5, 49). Finally, future work should continue the evaluation of the many interventions directed at frontline care delivery, health care organization, external environment, individual mediating factors to address clinician burnout and other adverse mental health outcomes.

## Conclusion

In conclusion, this study highlights COVID-19 and other pandemic-related challenges identified by physicians that have negatively impacted their mental health. Front line managers and health care systems can attenuate some of these stressors by providing additional targeted supports (i.e., sufficient testing supplies and PPE, childcare supports). However, the study also identifies specific sources of support–namely emotional, informational, appraisal, mental health resources and programs that build a resilient mindset–that can be impactful interventions that can be delivered by front line managers and at health system and organizational level.

## Data availability statement

The original contributions presented in the study are included in the article/supplementary material, further inquiries can be directed to the corresponding author.

## Ethics statement

The studies involving human participants were reviewed and approved by Northwell Health Institutional Review Board. The patients/participants provided their written informed consent to participate in this study. Written informed consent was obtained from the individual(s) for the publication of any potentially identifiable images or data included in this article.

## Author contributions

Conceptualization: RS, LR, SC, PS, MB, SJ, NP, and JY. Data curation: MW, LR, and KF. Formal analysis: MW, RS, SC, KF, and SJ. Investigation: MW, RS, SC, KF, SJ, NP, LR, and PS. Methodology: MW, RS, JY, SJ, SC, and KF. Project administration: RS, SC, SJ, and LR. Resources: RS, MW, and SJ. Software: SC and MW. Supervision: RS and SJ. Validation: MB, JY, and NP. Visualization: MW, KF, PS, and LR. Writing—original draft: MW, LR, PS, RS, SC, and SJ. Writing—review and editing: MW, LR, RS, PS, SC, MB, JY, NP, and SJ. All authors contributed to the article and approved the submitted version.
